# Interaction of bacterial genera associated with therapeutic response to immune checkpoint PD-1 blockade in a United States cohort

**DOI:** 10.1186/s13073-022-01037-7

**Published:** 2022-03-29

**Authors:** Rachel C. Newsome, Raad Z. Gharaibeh, Christine M. Pierce, Wildson Vieira da Silva, Shirlene Paul, Stephanie R. Hogue, Qin Yu, Scott Antonia, Jose R. Conejo-Garcia, Lary A. Robinson, Christian Jobin

**Affiliations:** 1 Department of Medicine, University of Florida College of Medicine, Gainesville, FL USA; 2 Department of Cancer Epidemiology, Moffitt Cancer Center, Tampa, FL USA; 3 Present Address: Department of Epidemiology, BARDS, MRL, Merck Sharp & Dohme Corp., a Subsidiary of Merck & Co., Inc., Kenilworth, NJ USA; 4 Department of Thoracic Oncology, Moffitt Cancer Center, Tampa, FL USA; 5 Department of Immunology, Moffitt Cancer Center, Tampa, FL USA; 6 Department of Infectious Diseases and Immunology, University of Florida College of Medicine, Gainesville, FL USA; 7 Department of Anatomy and Cell Biology, University of Florida College of Medicine, Gainesville, FL USA

**Keywords:** Microbiome, Immunotherapy, Non-small cell lung cancer, Fecal microbiota transplant

## Abstract

**Background:**

Recent studies show that human gut microbial composition can determine whether a patient is a responder or non-responder to immunotherapy but have not identified a common microbial signal shared by responding patients. The functional relationship between immunity, intestinal microbiota, and NSCLC response to immune checkpoint inhibitor/inhibition (ICI) in an American cohort remains unexplored.

**Methods:**

RNAlater-preserved fecal samples were collected from 65 pre-treatment (baseline) and post-treatment stage III/IV NSCLC patients undergoing ICI therapy, categorized as responders or non-responders according to RECIST criteria. Pooled and individual responder and non-responder microbiota were transplanted into a gnotobiotic mouse model of lung cancer and treated with ICIs. 16S rDNA and RNA sequencing was performed on patient fecal samples, 16S rDNA sequencing on mouse fecal samples, and flow cytometric analysis on mouse tumor tissue.

**Results:**

Responder patients have both a different microbial community structure than non-responders (*P* = 0.004) and a different bacterial transcriptome (PC2 = 0.03) at baseline. Taxa significantly enriched in responders include amplicon sequence variants (ASVs) belonging to the genera *Ruminococcus*, *Akkermansia*, and *Faecalibacterium*. Pooled and individual responder microbiota transplantation into gnotobiotic mice decreased tumor growth compared to non-responder colonized mice following ICI (*P* = 0.023, *P* = 0.019, *P* = 0.008, respectively). Responder tumors showed an increased anti-tumor cellular phenotype following ICI treatment. Responder mice are enriched with ASVs belonging to the genera *Bacteroides*, *Blautia*, *Akkermansia*, and *Faecalibacterium*. Overlapping taxa mapping between human and mouse cohorts correlated with tumor size and weight revealed a network highlighting responder-associated ASVs belonging to the genera *Colidextribacter*, *Frisingicoccus*, *Marvinbryantia*, and *Blautia* which have not yet been reported.

**Conclusions:**

The role of isolate-specific function and bacterial gene expression in gut microbial-driven responsiveness to ICI has been underappreciated. This work supports further investigation using isolate-driven models to characterize the mechanisms underlying this phenomenon.

**Supplementary Information:**

The online version contains supplementary material available at 10.1186/s13073-022-01037-7.

## Background

Recent advances in immunotherapy treatments have led to unprecedented survival rates among select patients with non-small cell lung cancer (NSCLC), particularly those with advanced-stage disease [1, 2]. Immune checkpoint inhibition (ICI) is one type of immunotherapy regimen which blocks immune tolerance pathways overexpressed by tumor cells, allowing the immune system components to remain activated against cancer cells. One type of immune checkpoint inhibitor is anti-programmed cell death receptor (PD)-1 (monoclonal antibody directed against inhibitory receptor PD-1) which seeks to inhibit the interaction between PD-1, which is expressed on the surface of immune cells, and programmed cell death ligand-1 (PD-L1) present on tumor cells. Despite recent success in clinical trials using immune checkpoint blockade, a minority (less than 20%) of NSCLC patients actually derive clinical benefit [3].

Of recent interest in this field is the impact of the human gut microbiome on cancer therapy. In 2018, one research group identified responsiveness to PD-1 by NSCLC patients as being associated with the enrichment of *Akkermansia muciniphila* in the intestinal microbiota [4]. More recently, Lee et al. identified *Bifidobacterium bifidum* as being enriched in patients responsive to therapy (ICI, chemotherapy, anti-EGFR) and *Akkermansia muciniphila* as being enriched in non-responders [5]. The opposite association of *Akkermansia muciniphila* with PD-1 responsiveness between cohorts highlights the complexity of microbiota interaction with therapeutics. Whether specific or broad genus- and species-level microbial abundance defines the NCSLC patient’s response to ICI remains unresolved. In this study, we used preserved fecal samples from a NSCLC cohort, multi-omics analysis, and humanized gnotobiotic mouse models to decipher, in more detail, the link between microbiota and responsiveness to ICI treatment.

## Methods

### Subject details and sample collection

Our study cohort comes from a prospective observational study that collected longitudinal stool samples from stage III/IV non-small cell lung cancer (NSCLC) patients receiving immune checkpoint inhibitor (ICI) treatment at Moffitt Cancer Center between June 2016 and February 2019. Patients who were prescribed treatment with ICI (single agent or in combination with other therapies) were invited to participate. Informed consent was obtained from patients once the study was approved by Advarra IRB (MCC#18611, Pro00017235). Each participant was provided an at-home fecal collection kit, with 8 mL of RNAlater preservative inside of a Sarstedt® fecal collection tube [6]. Additionally, a subset of participants also received and completed a Liquid Dental Transport Medium (LDTM; Anaerobe Systems, Morgan Hill, CA) stool collection kit meant to preserve bacterial viability for functional studies. Approximately 11 mL of LDTM was placed anaerobically inside of a Sarstedt® fecal collection tube and provided in a sealed pack. Fecal specimens were collected within 72 h (or 24 h, if LDTM was provided in the kit) of their clinic visit, with one kit being collected at the first dose of ICI treatment (baseline) and again after 4 doses of ICI (follow-up). Returned specimens were homogenized and stored at − 80 °C before downstream processing.

A total of 65 individual patients provided at least one stool sample for the study (Table 1). Fifty-eight (89%) patients were white, and 33 (51%) were male, with a median age of 66 and mean BMI of 26.1. The majority of patients (94%) presented with stage IV NSCLC as the primary diagnosis, and 92% of patients had a history of smoking. Eighteen (28%) subjects were categorized as responders to immunotherapy and 47 (72%) as non-responders using a two versus two response classification. Of the 18 responders, 15 (83%) received anti-PD-1, and three (17%) received anti-PD-L1. Of the 47 non-responders, 29 (62%) received anti-PD-1, 16 (34%) received anti-PD-L1, and two (4%) received a combination of anti-PD-L1/CTLA-4. Five (28%) responders and 13 (28%) non-responders received antibiotics prior to treatment, and many patients received prior or concurrent chemotherapy and/or radiation. Of the responding patients, the mean length of progression-free survival (PFS) was 514.7 days, with 65% of responders having a PD-L1-positive tumor. Of the non-responding patients, the mean length of PFS was 207.7 days, with 49% having a PD-L1-positive tumor. Cohort characteristics such as antibiotic treatment status, age, and gender were considered in all microbiota analyses. All 65 subjects’ fecal samples were subject to 16S rRNA gene sequencing analysi**s**. To rule out the possibility of the enrichment and depletion of amplicon sequence variants (ASVs) due to the differences in the patient’s characteristics, we performed differential abundance tests for each of these variables and excluded ASVs found to be significantly different in any of these tests. RNAseq analysis was performed on the fecal RNA of all patients with matched baseline Liquid Dental Transport Medium (LDTM)-preserved samples (*n* = 10) and a randomly selected subset of 10 additional baseline patient samples (Figure S2B). Again, we tested for the effect of the patient’s variables on sample clustering and differential expression and excluded any gene with *P*-value < 0.05 from further analyses.

**Table 1:** Overview of the study cohort characteristics. Patient characteristics stratified by response status for all 65 patients from Moffitt Cancer Center receiving ICI for stage III/IV non-small cell lung cancer. Values are represented in number and percentage or mean and standard deviation. Responder (R) vs. non-responder (NR) study cohort characteristics

	Total	% or SD	R	% or SD	NR	% or SD
Number of patients (total = 65)	65	100%	18	28%	47	72%
Liquid Dental Transport Medium (LDTM) stool	16	25%	4	22%	6	13%
Gender						
Male	33	51%	8	44%	25	53%
Female	32	49%	10	56%	22	47%
Race						
White	58	89%	17	94%	41	87%
Black or African American	6	9%	1	6%	5	11%
Others	1	2%	0	0%	1	2%
Antibiotics taken 2 months prior to treatment	18	28%	5	28%	13	28%
Pro/prebiotics taken 2 months prior to treatment	10	15%	3	17%	7	15%
NSCLC stage at ICI treatment start						
IIIA	3	5%	1	6%	2	4%
IIIB	1	2%	0	0%	1	2%
IV	61	94%	17	94%	44	94%
Type of immunotherapy given						
Anti-PD-1	43	66%	15	83%	29	60%
Anti-PD-L1	19	29%	3	17%	16	34%
Anti-PD-1, CTLA-4	2	3%	0	0%	2	4%
Tumor biopsy PDL-1 gene mutation test result						
Positive	35	54%	12	67%	23	49%
Negative	21	32%	4	22%	17	36%
Not tested	9	14%	2	11%	7	15%
Mean PLD-1 percent (if positive)	57%	± 35%	65%	± 34%	53%	± 36%
Adverse event related to treatment	53	82%	14	78%	39	83%
Prior/concurrent treatment						
Chemotherapy use prior to ICI	37	57%	8	44%	29	62%
Thoracic radiation prior to ICI	19	29%	5	28%	14	30%
Other radiation prior to ICI	24	37%	6	33%	18	38%
Targeted therapy prior to ICI	14	22%	2	11%	12	26%
Immunotherapy prior to ICI	3	5%	1	6%	2	4%
Concurrent chemotherapy	37	57%	11	61%	26	55%
Concurrent thoracic radiation	8	12%	1	6%	7	15%
Concurrent other radiation	14	22%	3	17%	11	23%
Baseline smoking history (current or former)	60	92%	17	94%	43	91%
Prior presence of colitis	8	12%	2	11%	6	13%
Prior presence of *H. pylori* infection	2	3%	1	6%	1	2%
Durable response						
Responder	22	34%	18	100%	0	0%
Non-responder	43	66%	0	0%	47	100%
Mean PFS	292.7	± 231.3	514.7	± 209.3	207.7	± 177.7
Mean age at presentation	66.2	± 8.9	64.7	± 7.0	66	± 9.7
Mean BMI	26.1	± 6.3	26.0	± 3.8	26.1	± 7.0

### Participant metadata collection

Clinical information was collected from the medical records, including tumor stage, histology and pathology (e.g., tumor diagnosis, mutation information), anthropometrics, and medication information (assessed at three defined time intervals: 6 months prior to, during, and up to 2 months after the start of ICI treatment). Antibiotic and pro-/prebiotic use extracted from the medical records was also supplemented with patient-reported use. Previous cancer treatments (i.e., chemotherapy, radiation, targeted therapy, and other immunotherapy) were also documented from 1 year prior to 1 year post-ICI treatment start. Patient demographics, smoking status (current, former, or never), were abstracted from MCC’s Electronic Patient Questionnaire (EPQ).

### Response assessment

A single radiologist evaluated all patients’ radiological images using RECIST v1.1 criteria to ensure consistency in response assignments regardless of patient participation in clinical trials or standard of care treatment. Clinical response was defined as a partial or complete response that persisted on at least 2 CT scans taken at least 1 month apart following ICI start, while non-response was defined as stable or progressive disease. Secondary outcomes included clinician- and patient-reported adverse events and were identified using the medical record and PRO-CTCAE, respectively. Progression-free survival was assessed via time to progression, death, or last contact.

### Mice

All animal experiments were approved by the Institutional Animal Care and Use Committee (IACUC) at the University of Florida (UF) and performed at UF Animal Care Facilities (IACUC Protocol #201909606). Colonies of germ-free mice were bred and maintained in isolators by UF Animal Care Services Germfree Division. Mixed gender germ-free wild-type (GF-WT) C57BL/6 mice were transferred from breeding isolators and placed into the Techniplast ISOcage P Bioexclusion system to allow for microbial manipulation [7, 8]. Cages were sterilely changed every 2 weeks per Techniplast protocol and supplied with autoclaved food and water.

### Fecal microbiota transplantation

Homogenized LDTM-preserved patient samples were individually thawed, each placed into an anaerobic chamber for no more than 90 s, and pooled by response phenotype (R: *n* = 4, NR: *n* = 6) at an average anaerobic CFU/mL concentration of 5 × 10^7^ CFU/mL as estimated by anaerobic CFU assay. Pooled samples were gavaged into mixed gender GF-WT mice (pre-treatment/responder and pre-treatment/non-responder) for comparison to untreated GF-WT mice (*n* = 9/group). For individual subject fecal microbiota transplant (FMT) studies, subjects R-139, R-134, NR-126, and NR-135 were selected from the original pooled samples for the same experiment as described before, but the LDTM-preserved fecal samples were directly gavaged at 10^7^ CFU/mouse (*n* = 9/group).

### Cell lines

The Lewis lung carcinoma cell line LL/2 (LLC1) (ATCC® CRL-1642™) was obtained from the American Type Culture Collection (ATCC) and transfected with ready-to-use lentiviral particles expressing firefly luciferase with GFP and puromycin dual markers (GenTarget). Following puromycin selection, luciferase production was confirmed using the Pierce Firefly Luciferase Glow Assay Kit (Thermo Fisher). Cells were cultured in Dulbecco’s modified Eagle’s medium, supplemented with fetal bovine serum (ATCC) to a final concentration of 10% and 1% penicillin/streptomycin, and maintained in a humidified incubator at 37 °C and 5% CO_2_.

### Human fecal samples RNA and DNA isolation

Human fecal samples preserved in RNAlater were extracted using the PowerLyzer® PowerSoil® DNA Isolation Kit (QIAGEN). Briefly, each tube of RNAlater preserved feces was completely homogenized and aliquoted post-collection and prior to freezing. These homogenized aliquots were placed one at a time into the biosafety cabinet to slightly thaw to allow removal of 100–200 mg of frozen fecal slurry, which was directly placed into a 1.7-mL sterile Eppendorf tube. This tube was centrifuged at 10,000 RPM for 5 min. The RNAlater supernatant was removed, and 50–70 mg of stool was promptly added to a PowerLyzer® Glass Bead Tube, to which 750 μL of Bead Solution was added and a standard kit procedure was used to isolate the DNA. For DNA isolation from fecal samples preserved in LDTM (used in the fecal transplant experiments in mice), the same procedure was followed, with the modification that the sample was completely homogenized in LDTM prior to removal of a sample for isolation. As before, this sample was directly placed into a 1.7-mL sterile Eppendorf tube and centrifuged at 9600×*g* for 5 min before processing of the stool sample with the PowerLyzer® PowerSoil® DNA Isolation Kit (QIAGEN). All samples were randomly assigned to extraction batches, with each having a negative control (extraction blank). Additionally, positive and negative PCR controls were used for barcoding, along with both ZymoBIOMICS microbial community and microbial community DNA standards. A second aliquot was taken from the same tube as the sample for DNA extraction but was instead processed using the mirVana miRNA isolation kit (Fisher/Ambion). Briefly, 50–70 mg of stool sample was placed into a screw cap tube with a ~ 250-μL 1:1 mix of 1 mm acid-washed glass beads and 0.1 mm zirconia beads. Immediately, 700 μL of mirVana lysis/binding buffer was added to the tube, and the samples were homogenized by bead beating at 5000 rpm for 3 × 30 s (Precellys 24). Following homogenization, the samples were processed following the standard kit instructions. RNA samples were DNases-treated using the Turbo DNA-free kit (Ambion) and then processed as described below for RNA sequencing.

### Tumor challenge and treatment

Two weeks post-inoculation, ISOcage P Bioexclusion cages were sterilized with Exspor, and the mice were implanted subcutaneously with 10^6^ Lewis lung carcinoma cells expressing a luciferase reporter (LLC-luc) inside a sterilized biosafety cabinet. Allograft implantation, injections, and all measurements were performed using sterile instruments for each mouse in a laminar flow hood following sterilization of the work surfaces with Exspor. Freshly passed stools were collected using autoclaved forceps into sterile Eppendorf tubes and snap-frozen before gavage, and then weekly following the start of anti-PD-1 antibody injection until the end of the experiment using the same sterile technique. The biosafety cabinet was sterilized completely along with all cages in between groups of mice. Following manual palpation detection of the tumor, mice were injected intraperitoneally with anti-PD-1 mAb (250 μg; clone RMP1-14, BioXcell) or untreated (control) every 3 days until an average tumor size for any group reached the size criteria for euthanasia as determined by the Institutional Animal Care and Use Committee (IACUC) guidelines (greater than 1.5 mm^3^). For each injection and/or measurement, all cages and work surfaces including manual calipers were sterilized via the Exspor protocol. Tumor size was measured via Exspor-sterilized manual caliper measurement every 2–3 days following implantation. At the endpoint, mice were imaged for tumor visualization using the IVIS imaging system, which was performed under SPF conditions, followed by immediate euthanasia and dissection.

### Murine fecal DNA isolation and 16S rRNA gene sequencing and analysis

One-half murine fecal pellet per mouse was aliquoted into a 96-well PowerBead Pro Plate (QIAGEN), and samples were extracted using the DNeasy 96 PowerSoil Pro QIAcube HT (QIAGEN). Briefly, samples were homogenized in lysis buffer using the TissueLyser II (QIAGEN) before subsequent processing by the QIAcube HT as previously described [9]. Following fecal DNA extraction, the 16S rRNA gene V1–V3 hypervariable region was amplified using barcoded primer pairs 27F (5 = -AGAGTTTGATCCTGGCTCAG-3 =) and 534R (5 = -ATTACCGCGGCTGCTGG-3 =) with universal Illumina paired-end adapter sequences. PCR products were purified, quantified, and pooled as described previously and sequenced with an Illumina MiSeq [10]. Patient RNAlater samples were sequenced using one run of Illumina MiSeq (2 × 300), and fecal microbiota transfer samples (mouse and human LDTM inoculum samples) were sequenced in another Illumina MiSeq (2 × 300) run. Sequencing reads were preprocessed using Quantitative Insights into Microbial Ecology version 2 (QIIME2) [11] including trimming, filtering at Q20 and pair merging. The final set of reads was fed to the DADA2 algorithm within QIIME2 pipeline to infer exact amplicon sequence variants (ASVs) with trim length set to 200 (the average sequence length of the dataset) [12].

The human dataset contained an average of 56,238 reads per sample (min = 10,871 reads; max = 85,121 reads) incorporated in ASVs, and the mouse dataset contained an average of 57,478 reads per sample (min = 30,588 reads; max = 95,926 reads) incorporated in ASVs. For the mouse dataset, we intersected the human inoculum ASVs and the mouse ASVs and used the resulting set, which has an average of 38,796 reads (min = 8,803 reads; max = 65,109 reads), in all subsequent analyses. Taxonomic assignment was done using QIIME2’s feature-classifier classify-sklearn after training the classifier on the SILVA reference dataset (v.138). Only ASVs with bacterial taxonomy were used in subsequent analyses.

We generated principal coordinate analysis (PCoA) using the phyloseq R package [13] from weighted UniFrac [14] distance metric after rarefication to the minimum read count in each dataset. The difference in the microbial community composition (beta diversity) due to response status or patient’s characteristics was tested using permutational multivariate analysis of variance (PERMANOVA) through the vegan R package command adonis (version 2.5) with permutations set to 1000 for the human data and confirmed with R’s generalized least squares linear model (gls). For the mouse data, the differences were tested as described previously [15, 16]. Briefly, we used a linear mixed-effects model (lme function) in the R nlme package (version 3.1-140), with the REML method to fit a generalized mixed linear model of the following form: PCoA ~ status + 1|cage + *ɛ*, where status indicates responder or non-responder and 1|cage indicates that we used the cage as a random effect to account for cohousing effects.

Differential abundance analysis was performed using edgeR [17] through phyloseq to_edgeR function at the ASV level. We considered an ASV differentially abundant if its edgeR FDR-corrected *P*-value is less than 0.05. To rule out the possibility of the enrichment and depletion of ASVs due to the differences in the patient’s characteristics, we performed differential abundance tests for each of these variables and excluded ASVs found to be significantly different in any of these tests.

### Microbial RNA sequencing and analysis

Quality control, rRNA depletion, and cDNA library preparation were performed by the UF’s Interdisciplinary Center for Biotechnology Research (ICBR) Gene Expression and Genotyping core using the Agilent 2100 bioanalyzer (Agilent Genomics catalog no. G2939BA), Ribo-Zero Gold rRNA removal kit (Epidemiology) (Illumina catalog no. MRZE724), and ScriptSeq v2 RNASeq library preparation kit (Illumina catalog no. SSV21124) starting with 1 μg total RNA. Samples were sequenced by the UF ICBR NextGen DNA Sequencing core using one lane of Illumina HiSeq 3000 (2 × 100). Reads were quality trimmed and filtered (at Q20) to remove human (using iGenome *Homo sapiens* GRCh38 reference genome) rRNA and tRNA (using a collection of NCBI rRNA and tRNA in addition to SILVA database sequences) by employing Trimmomatic (v.0.36) [18] and KneadData (http://huttenhower.sph.harvard.edu/kneaddata). The quality trimmed and filtered dataset contained an average of 43,043,858 reads (min = 3,469,691 reads; max = 82,543,054 reads). Next, we generated a de novo reference metagenome assembly from Routy et al. [4] non-small cell lung cancer (NSCLC) shotgun fecal samples (after trimming and filtering the reads as described above) using MEGAHIT (v.1.2.8) [19] and annotated it using prokka (v.1.12) [20]. The trimmed and filtered RNAseq reads were then aligned to the NSCLC reference metagenome using Bowtie2 (v.2.3.5) [21] followed by quantification using featureCounts from the subread package (v.1.5.3) [22].

Output from featureCounts was imported to edgeR (v.3.26) [17] for normalization, principal component analysis (PCA), and differential expression analysis. We considered a transcript differentially expressed if its edgeR FDR adjusted *P*-value < 0.05. We also tested for the effect of the patient’s variables on sample clustering and differential expression and excluded any gene with *P*-value < 0.05 from further analyses. Pathway analysis was conducted through GAGE (v.2.34) [23] using the Kyoto Encyclopedia of Genes and Genomes (KEGG) reference pathways after the trimmed and filtered reads were aligned to a local copy of KEGG bacterial orthologs using Diamond aligner (v.0.9) [24]. We considered a pathway significant if its GAGE FDR-P is less than 0.05.

### Cytoscape network

The network was generated using Cytoscape (version 3.8.2) (https://cytoscape.org/) from a table of significant correlation between significantly enriched taxa and mouse tumor size/weight in addition to edgeR log_2_ fold changes of these significantly enriched taxa [25]. The correlations were generated using Pearson correlation through R’s cor.test function between mouse tumor size/weight and taxa abundances, and only those with *P*-value < 0.05 were used.

### Flow cytometry

Following the excision of allograft tumors from mice at the endpoint, the tumors were finely divided using a surgical blade and kept at 4 °C in PBS buffer containing 2 mg/mL Stemxymel (Worthington Biochemical Corp.) and 0.1 mg/mL DNase I (StemCell Technologies). Tissues were then incubated at 37 °C for 15 min and transferred to 25C tubes (Miltenyi Biotec) for homogenization by the gentleMax dissociator (Miltenyi Biotec). Dissociated tissue was then passed through a 70-μm cell strainer by centrifugation at 300*g* for 5 min. The cell pellet was washed twice and resuspended in cold cell staining buffer (Biolegend). Following dissociation of the tumor to single cells, LIVE/DEAD Fixable Violet Dead Cell Stain Kit (Thermo Fisher) was added to the pelleted cells and stained for 15 min at 4 °C. Cells were then stained with cell surface antibodies for flow cytometry analysis: CD45 BV510 (Biolegend, clone 30-F11), CD4 BV711 (Biolegend, clone GK1.5), CD8 PE (Biolegend, clone 53-6.7), CD3 BV605 (Biolegend, clone 17A2), CXCR3 APC-Cy7 (Biolegend, clone CXCR3-173), F4-80 BV605 (Biolegend, clone T45-2342), Gr-1 BV711 (Biolegend, clone RB6-8C5), CD11b APC-Cy7 (Biolegend, clone M1/70), and CD11c-PE (Biolegend, clone N418). To quantify IFNγ produced by T cells, the dissociated single-cell suspensions were incubated with 1× Brefeldin A solution (Biolegend) and cell stimulation cocktail (Thermo Fisher) for 4 h prior to harvesting and staining. Following cell surface marker staining, cells were washed in staining buffer as before and then permeabilized and fixed using a fixation/permeabilization kit (BD Biosciences). Following permeabilization, for IFNγ production, cells were stained with IFNγ PE-Cy7 (Biolegend, clone XMG1.2). Flow cytometry was performed on a BD LSR Fortessa flow cytometer in the UF ICBR (BD Biosciences) and analyzed using the FlowJo software version 10.6.1. Gating strategies for IFNγ production, myeloid, and T cell activation panels are shown (Figure S4A and S4B and S4C, respectively).

### Statistical analysis

Statistical comparison between the two groups was performed using a two-tailed Mann-Whitney *U*-test while multi-group comparisons were performed by one-way or two-way ANOVA, where appropriate, using GraphPad Prism 6. Statistical tests for each *P*-value are indicated in the text. *P*-values < 0.05 were considered statistically significant, and all *P*-values were reported to three significant figures.

## Results

### Patients characteristics at baseline

Our study cohort comes from a prospective observational study that collected longitudinal stool samples from stage III/IV non-small cell lung cancer (NSCLC) patients receiving immune checkpoint inhibitor (ICI) treatment at Moffitt Cancer Center (MCC#18611) (see the “Methods” section).

### Responders to immunotherapy have a different microbial community structure and transcriptome than non-responders at baseline

**Fig. 1:**
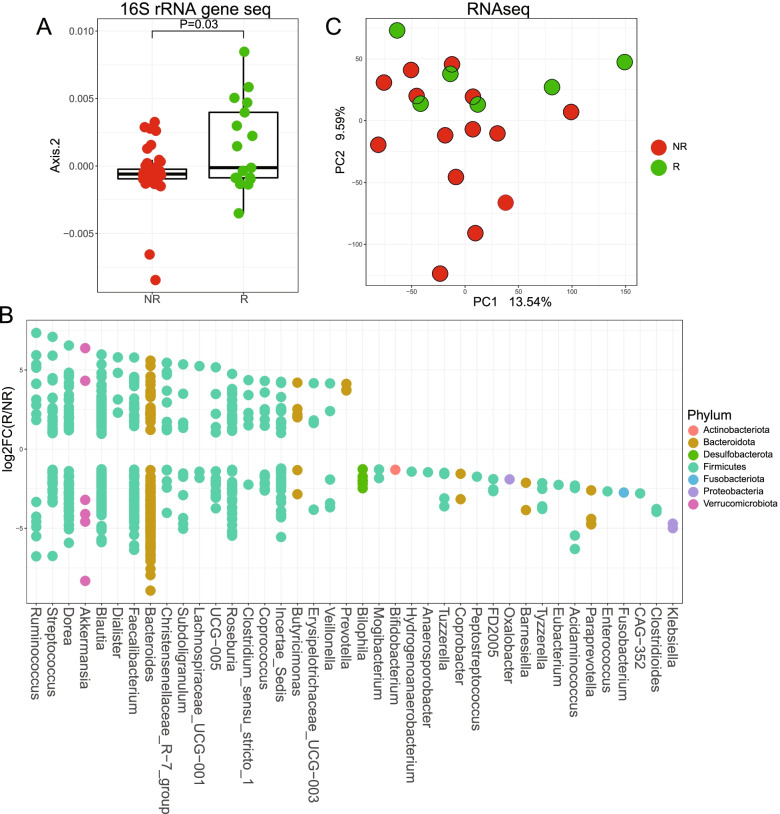
Responders to immunotherapy have a different microbial community structure and metatranscriptome than non-responders at baseline. **A** Box plot of weighted UniFrac second PCoA of responder (*n* = 22) and non-responder (*n* = 43) participants showing the difference between the two groups. PERMANOVA *P* = 0.03, gls *P* = 0.004 (see Additional file 7: Figure 1A for full PCoA). **B** Log_2_ fold change (log2FC) plot of significantly (FDR-P < 0.05) enriched amplicon sequence variants (ASVs) in responder versus non-responder subjects. Filled circles located above *y* = 0 indicate enrichment in responders, and those below indicate enrichment in non-responders. Only significant ASVs for the top 20 enriched genera in each direction are shown. See Additional file 7: Figure S1B for the full list. **C** PCA showing the different clustering of responders (*n* = 6) versus non-responders (*n* = 14) metatranscriptomes. gls *P* = 0.032

At baseline, the microbial community structure of patients responding to the therapy is significantly different than non-responders (Fig. 1A and Additional file 1: Figure S1A). The analysis of the log fold change of significantly different taxa between responders and non-responders revealed an enrichment in amplicon sequence variants (ASVs) belonging to the genus *Ruminococcus* as the strongest associated taxa for response (Fig. 1B and Additional file 1: Figure S1B). Additional enriched taxa in responders include ASVs belonging to the genera *Akkermansia*, *Blautia*, and *Faecalibacterium*; however, most enriched responder genera have ASVs that are also enriched in non-responders. When examining the potential confounding variables, there was no significant effect of the treatment regimen, age, gender, disease progression, and other collected variables (Table 1) on response. Most notably, exposure to antibiotics before treatment had no effect on sample clustering.

To expand our microbial analysis to function associated with ICI treatment, we performed RNAseq analysis on the fecal RNA of all patients with matched baseline Liquid Dental Transport Medium (LDTM)-preserved samples (*n* = 10) and a randomly selected subset of 10 additional baseline patient samples (Additional file 2: Figure S2B). RNAseq analysis showed that the bacterial transcriptome is also significantly different between responders and non-responders at the baseline collection time point (Fig. 1C and Additional file 2: S2A). Thirty genes were significantly upregulated in responder patients, and 10 genes were significantly upregulated in non-responder patients (Additional file 6: Table S1). The microbial pathways that are enriched in responders include carbon fixation pathways in prokaryotes, while phosphotransferase systems were enriched in the non-responders (Additional file 2: Figure S2C Responders and S2D Non-Responders). Taken together, these data indicate differential gut microbiota configuration between responders and non-responders undergoing anti-PD-1/PD-L1 therapy.

### Responder microbiota transplantation decreased tumor growth compared to non-responder colonized mice following immunotherapy treatment

**Fig. 2:**
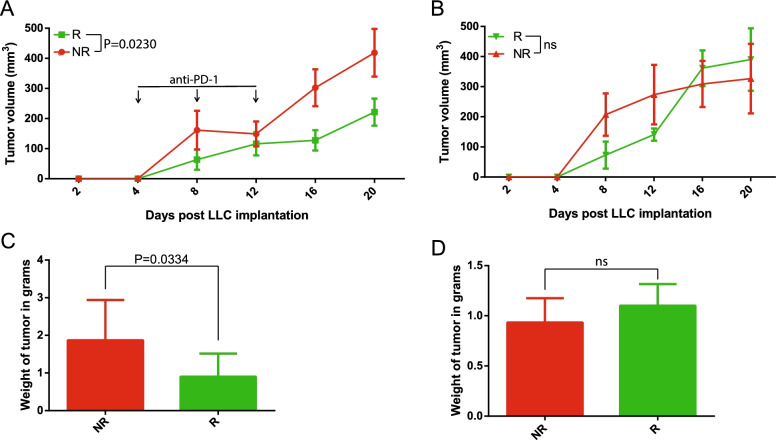
Responder microbiota transplantation decreases tumor growth compared to non-responder colonized mice following immunotherapy treatment. **A** Growth curve of LLC-luc subcutaneous allograft tumors after human fecal microbiota transplant from responder (*n* = 4) or non-responder (*n* = 6) pooled feces into germ-free mice (*n* = 9/group) treated with anti-PD-1 monoclonal antibody injection. Each point is tumor volume mean ± SEM. ANOVA *P* = 0.023 at the endpoint. **B** Growth curve of untreated LLC-luc subcutaneous allograft tumors after human fecal microbiota transplant from responder (*n* = 4) or non-responder (*n* = 6) pooled feces into germ-free mice (*n* = 5/group). Each point is tumor volume mean ± SEM. ANOVA *P* > 0.05 at the endpoint. **C** Mean ± SEM of tumor weight at the endpoint for mice treated with an anti-PD-1 monoclonal antibody that received FMT from either responder or non-responder pooled feces. Mann-Whitney *P* = 0.033. **D** Mean ± SEM of tumor weight at the endpoint for untreated mice that received FMT from either responder or non-responder pooled feces. Mann-Whitney *P* > 0.05

To establish the relationship between microbial composition and ICI response in a preclinical model of lung cancer, LDTM-preserved patient samples were pooled by response phenotype (R: *n* = 4, NR: *n* = 6) and gavaged into germ-free wild-type (GF-WT) mice (C57BL/6, *n* = 9/group) housed in ISOcage P Bioexclusion system to prevent bacterial contamination from the external environment. Two weeks post-colonization, mice were implanted with Lewis lung carcinoma cells (LLC-luc) and either treated with anti-PD-1 or untreated (control). Mice colonized with responder biota showed decreased tumor growth and tumor weight compared to mice colonized with non-responder microbiota following anti-PD-1 treatment (Fig. 2A, C). There was no difference in tumor growth or weight in untreated mice that were colonized with the human microbiota (Fig. 2B, D). IVIS imaging (InVivo Imaging System) of the mice with luciferase-expressing tumors prior to euthanasia also demonstrated a significant difference in size and spread of tumors following treatment (Additional file 3: Figure S3A and S3B). The tumors in responder mice weighed significantly less than non-responder tumors (Fig. 2C).

We next used single patient fecal transfer to ascertain the responder phenotype. Participants R-139, R-134, NR-126, and NR-135 were selected from the original pooled samples for the same experiment as described before (*n* = 9/participant). At the endpoint, tumor volume was again significantly smaller in both R-139- and R-134-colonized mice (Figure S3C and S3D). The excised tumor weight was also significantly smaller in R-139 and R-134-colonized mice (Figure S3E and S3F). Overall, the responder microbiota from either pooled or individual samples confirmed the synergistic anti-PD-1 tumor effect with microbiota.

### Responder colonized mice show anti-tumor immune phenotype following treatment with immunotherapy

**Fig. 3:**
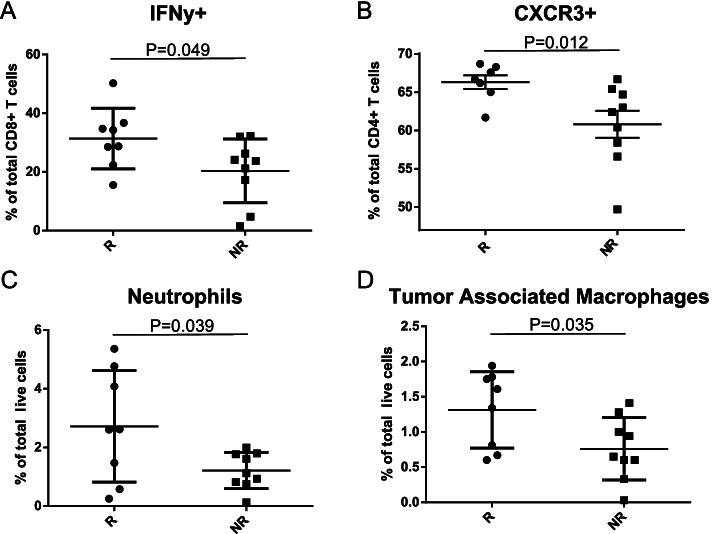
Responder colonized mice show anti-tumor immune phenotype following treatment with immunotherapy. Half of the resected subcutaneous allograft tumors from human microbiota colonized responder and non-responder mice (*n* = 9/group) were subjected to single-cell dissociation and flow cytometric analysis for **A** tumoral CD8+ IFNγ+ T cells as represented by percent of total intra-tumoral CD8+ T cells in responder (*n* = 8) and non-responder (*n* = 9) tumors, Mann-Whitney *P* = 0.049; **B** tumoral CD4+ CXCR3+ T cells as represented by percent of total CD4+ T cells in responder (*n* = 7) and non-responder (*n* = 9) tumors, Mann-Whitney *P* = 0.012; **C** tumoral neutrophils (Gr1+ CD11c+ CD11b+ cells) as represented by percent of total live cells in responder (*n* = 8) and non-responder (*n* = 9) tumors, Mann-Whitney *P* = 0.039; and **D** tumoral macrophages (Gr1− CD11c− CD11b+ cells) as represented by percent of total live cells in responder (*n* = 8) and non-responder (*n* = 9) tumors, Mann-Whitney *P* = 0.035

To examine the differences in immune tumor microenvironment between responder and non-responder colonized mice, tumors were resected from the pooled inoculum mice at the endpoint, finely divided, and aliquoted for flow cytometric analysis (Additional file 4: Figure S4). Tumor resident cytotoxic CD8+ IFNγ+ T cells were increased in the responder-colonized mice than non-responder mice (Fig. 3A and Additional file 5: Figure S5A), while no differences in the relative abundance of CD8+ or CD4+ T cell populations was found (data not shown). Furthermore, responder tumors had significantly more CD4+ T cells expressing the chemokine receptor CXCR3 (Fig. 3B and Additional file 5: Figure S5B). Lastly, the myeloid cell compartment of the tumor microenvironment was assessed, revealing that the population of both intra-tumoral neutrophils and tumor-associated macrophages (TAMs) was increased in responder-colonized tumors (Fig. 3C, D, Additional file 5: Figure S5C and Figure S5D). Taken together, these data indicate that the tumor immune microenvironment of responder microbiota-colonized mice displayed an anti-tumor cellular phenotype following anti-PD-1 treatment compared to non-responder microbiota colonized mice.

### The microbiota of humanized responder mice is different from that of non-responders

**Fig. 4:**
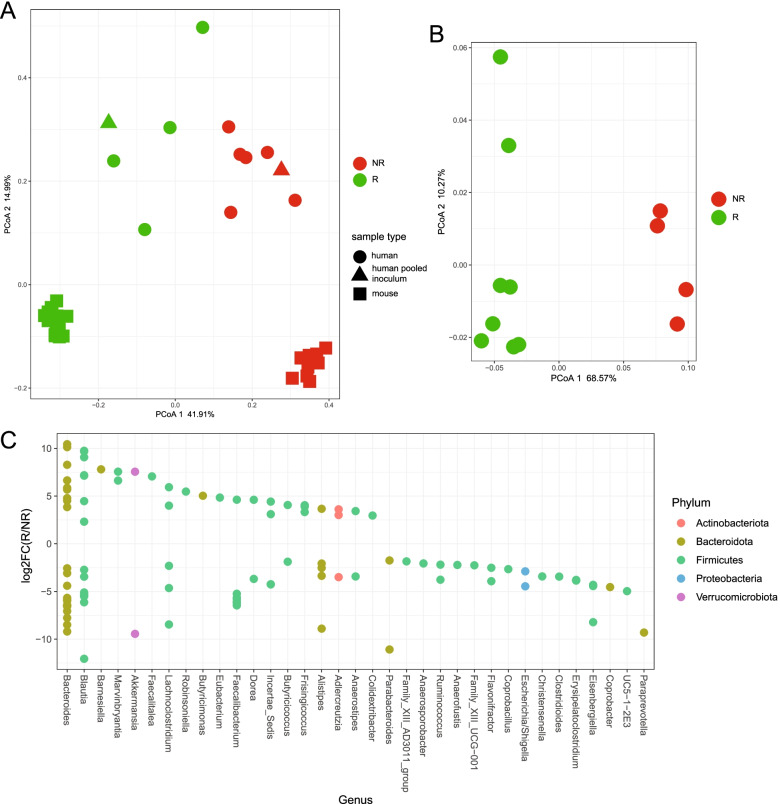
The microbiota of humanized responder mice is different from that of non-responders. 16S rDNA sequencing was performed on the feces collected from the anti-PD-1-treated pooled microbiota mice 2 weeks post-colonization and prior to the initial treatment, as well as individual patient inoculum microbiota and their associated pools. **A** Principal coordinates analysis (PCoA) showing beta diversity measured by weighted UniFrac distance between individual human donors (R: *n* = 4; NR: *n* = 6), pooled inoculums (R: *n* = 1; NR: *n* = 1) and mouse feces 2 weeks post-colonization with pooled donor inoculums and at endpoint (R: 2 weeks *n* = 8, endpoint *n* = 9; NR: 2 weeks *n* = 4, endpoint *n* = 8). **B** Principal coordinates analysis (PCoA) showing beta diversity measured by weighted UniFrac distance between individual mouse feces 2 weeks post-colonization with pooled donor inoculums (R: *n* = 8; NR: *n* = 4) lme *P* = 0.001. **C** Log_2_ fold change (log2FC) plot of significantly (FDR-P < 0.05) enriched ASVs in responder versus non-responder mice 2 weeks post-colonization with human pooled inoculums. Filled circles located above *y* = 0 indicate enrichment in responders, and those below indicate enrichment in non-responders

To connect the responder phenotype-associated bacterial taxa in both our human and mouse study cohorts, 16S rDNA sequencing was performed on the feces collected from the anti-PD-1-treated pooled microbiota mice 2 weeks post-colonization and prior to the initial treatment using ASVs present in the human inoculum only (see the “Methods” section). The microbial community structure of individual patient’s microbiota as well as their associated pooled microbiota was different between responder and non-responder subjects (Fig. 4A). Importantly, microbial community difference observed in this inoculum was even more pronounced following implantation in mice (Fig. 4B).

Of the 398 ASVs originally found in the responder human inoculum, 252 were found in the colonized mice 2 weeks post-gavage, for a 63% transference rate. Of the 433 ASVs found in the non-responder human inoculum, 230 were found in the non-responder colonized mice post-gavage, for a 53% transference rate. Analysis of the log_2_ fold change of significantly enriched taxa between responders and non-responders revealed ASVs belonging to the genus *Bacteroides* as being the most enriched in responders (Fig. 4C). Additional taxa enriched in the responder-colonized mice included ASVs belonging to the genera *Blautia*, *Akkermansia*, and *Faecalibacterium*, which were also significantly enriched in the responder patients.

**Fig. 5:**
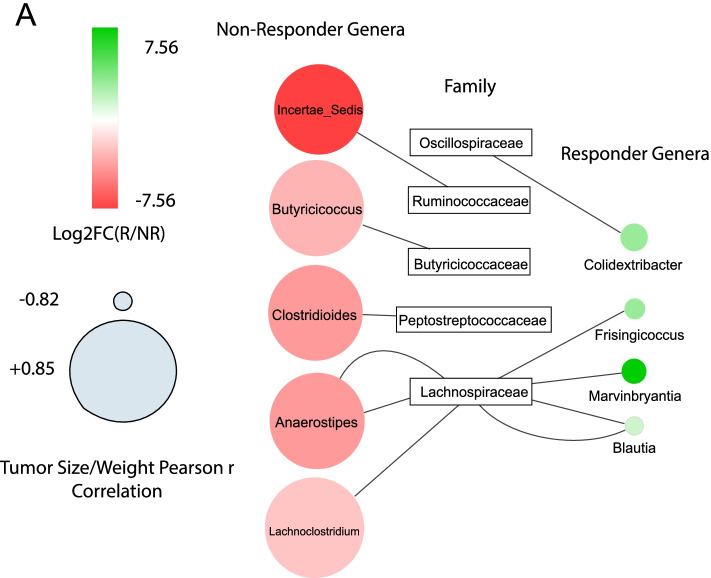
Common taxa between human inoculum and mouse cohorts correlated with tumor size or weight revealed a network highlighting ASVs belonging to responder-associated genera. **A** Taxa significantly enriched (FDR-P < 0.05), in mouse responders versus non-responders significantly correlating with either an increase or decrease tumor size or weight, were imported into Cytoscape (version 3.8.2) (https://cytoscape.org/) to generate a visual network of interactions between the nodes (genera) and edges (enrichment in mouse responders or non-responders, tumor size and tumor weight) [25]

Lastly, a map of response phenotype correlated with mouse tumor size/weight and significantly enriched taxa was generated to identify the most important ASVs belonging to genera for response (Fig. 5A). To generate these interactions, we took the overlapping significantly enriched ASVs in either responder or non-responder patients and correlated the same ASVs with resulting tumor size and weight in recipient mice undergoing anti-PD-1 therapy. The resulting map of interactions presents overlapping ASVs belonging to the genera significantly associated with response phenotype in both human and mouse datasets. The bacterial family Lachnospiraceae has the most ASVs associated with different genera, with three ASVs belonging to the family being associated with non-response and four ASVs being associated with response. The four taxa highlighted as being the overlapping signature of response are *Colidextribacter*, *Frisingicoccus*, *Marvinbryantia*, and *Blautia*. The five taxa highlighted as being the overlapping signature of non-response are an *Incertae sedis* genus from the family Ruminococcaceae, *Butyricicoccus*, *Clostridioides*, *Anaerostipes*, and *Lachnoclostridium*. Overall, our data linked novel fecal bacterial ASVs belonging to the genera with patients’ responsiveness to anti-PD-1 treatment.

## Discussion

Using patient fecal samples collected from participants undergoing ICI in a United States cohort, we found that responders and non-responders to ICI have a different gut microbial composition and transcriptome at baseline. Using functionally preserved fecal samples from a subset of patients, gnotobiotic mice were colonized with the feces of responding or non-responding patients, and maintained the response phenotype to anti-PD-1 treatment from their original donors, in a model of lung cancer traditionally resistant to ICIs [26]. By examining the overlapping microbial composition of the human and mouse cohorts, and correlating significantly enriched taxa with tumor size and weight in the mice, a group of novel taxa associated with response was identified.

Previous bodies of work have identified non-overlapping microbial signatures of response in NSCLC patients. Routy et al. was the first to examine the gut microbial composition of NSCLC responders and non-responders to ICI, using a cohort of 140 patients [4]. Enrichment of *Akkermansia muciniphila* was identified as the strongest signature of response, along with *Ruminococcus* spp. and *Alistipes* spp. Routy et al. also found that responder-colonized mice had tumors enriched in CXCR3+ CD4+ T cells, which overlaps with our tumor immune profile [4]. This receptor is associated with Th1-type CD4+ T helper cell function and is important for trafficking of T cells, indicating increased T cell movement and effector cell function in responder-colonized mice undergoing anti-PD-1 treatment [27]. In addition, the tumor myeloid cell compartment in our responders showed an increase in both neutrophils and tumor-associated macrophages (TAMs). Both intratumoral neutrophils and TAMs are generally considered pro-tumorigenic due to their immunosuppressive function, but there is evidence for their support in response to immune checkpoint blockade [28]. Generally, TAMs are associated with an immunosuppressive tumor microenvironment because of their function in promoting tumor-associated inflammation and also in increasing immune checkpoint protein expression in tumor cells. The latter function could be important for immune checkpoint blockade efficacy as these interactions are blocked by anti-PD-1 therapy.

Although an enrichment of *Akkermansia muciniphila* was identified to be the strongest signature of PD-1 responder by Routy et al., a recent study by Lee et al. using a cohort of 96 NSCLC patients observed that *Bifidobacterium bifidum* was more enriched in responders to therapy overall and interestingly that *Akkermansia muciniphila* and *Ruminococcus* were significantly enriched in non-responders [5]. Although only 22 of the 96 patients were treated with ICI, these differences highlight the lack of clarity regarding the microbial signature associated with responsiveness, and perhaps differences in the methodologies for collecting and processing specimens. In our cohort, responders to ICI have an enrichment of ASVs belonging to the genera *Ruminococcus* spp., as well as ASVs belonging to the genera *Akkermansia*, *Blautia*, and *Faecalibacterium*. Interestingly, however, each genus with ASVs significantly enriched in responders has ASVs that are significantly enriched in non-responders. The presence of oppositely enriched ASVs within each genus indicates that relative abundance at the genus level may not be detailed enough to identify a microbial signature associated with response.

In a meta-analysis of three landmark papers reporting microbiota relationship with PD-1 response efficacy, the microbial gene content of bacteria in patient stool samples was a more effective predictor of patient response than the abundance of specific bacteria alone, indicating that the mechanism behind bacterial-driven response may be more complex than originally thought [29]. Taken together, the unique functionality of specific isolates may be driving response phenotype in patients, and isolating and investigating these patient-derived strains in preclinical models as opposed to standard commercial vendors (e.g. ATTC, DSMZ) may add clarity to the relationship between microbes, immunity, and cancer therapeutics.

In the mouse cohort, taxa enriched in responders are primarily from ASVs belonging to the genus *Bacteroides*, whereas *Ruminococcus* is significantly enriched in non-responding mice. It is important to note that only 63% and 53% of taxa from human responder and non-responders, respectively, were able to engraft into the mouse model 2 weeks post-inoculation. Despite losing more than ~ 37% of the original human microbiota inoculum, mice transplanted with feces from PD-1-responding patients still transferred anti-tumor response following treatment. Moreover, by examining the overlapping microbiota between original human and transplanted mice and correlating enriched taxa with tumor size and weight from the mouse experiment, we identified novel ASVs belonging to genera associated with both responders and non-responders. The four taxa highlighted as being enriched in responder patients and mice and correlated with decreased tumor growth (*Colidextribacter*, *Frisingicoccus*, *Marvinbryantia*, and *Blautia*) are relatively unexplored in the area of immunotherapy response. However, the presence of one taxon, *Blautia*, has been associated with reduced mortality from graft-versus-host disease and has been shown to have anti-inflammatory activity beneficial in colorectal cancer [30, 31]. The five taxa highlighted as being enriched in non-responder patients and mice and correlated with increased tumor growth (ASVs belonging to the genera *Incertae sedis* from the family Ruminococcaceae, *Butyricicoccus*, *Clostridioides*, *Anaerostipes*, and *Lachnoclostridium*) also have no published association with ICI response in NSCLC. The current efforts are being devoted to isolating and characterizing the function of strains from each of these genera and other taxa of interest.

Additionally, microbial drivers of non-response were recently highlighted in a study by Andrews et al. which found microbial differences associated with increased adverse immune-related events following treatment with ICIs [32]. Although this was a cohort of melanoma patients, who clinically have twice the response rate to ICIs compared to NSCLC patients, the possibility of identifying and targeting taxa driving non-response is intriguing, and further work will be required to characterize the non-responder associated taxa from our cohort as well.

## Conclusions

Our work identifies a novel microbial signature associated with PD-1 responsiveness in NSCLC. Further work will be necessary to precisely establish the mechanisms through which these microorganisms modulate therapeutic response. Potential therapeutic avenues can then be explored based on these discoveries, including formulation of a designer microbe cocktail or precision dietic to produce optimal gut microbiota composition and function for the best response to ICIs [33]. Additionally, further investigation into the interaction of the gut microbiome with other immunotherapies such as CAR-T cell therapy, dendritic cell vaccines, and adoptive cell transfer, or even other cancer types, may demonstrate other avenues for improvement in therapeutic response.

## Supplementary Information

**Additional file 1: Fig. S1.** PCoA of baseline responder versus non-responder subjects, and full log fold change plot of all significantly enriched amplicon sequence variants (ASVs).

**Additional file 2: Fig. S2.** PCoA of RNAseq samples, Volcano plot visualization of gene expression for R versus NR subjects, and word cloud representation of functional pathways enriched in R and NR subjects.

**Additional file 3: Fig. S3.** Endpoint IVIS imaging and quantification and single donor FMT experiment tumor growth.

**Additional file 4: Fig. S4.** Representative flow cytometry gating strategies.

**Additional file 5: Fig. S5.** Quantitative representation of flow cytometric analysis.

**Additional file 6: Table S1.** Significantly upregulated pathways in responder and non-responder human subjects by RNAseq analysis.

**Additional file 7.** Supplementary Figure Legends.

## Data Availability

Sequencing data has been deposited in the National Center for Biotechnology Information (NCBI) Sequence Read Archive (SRA) under IDs: PRJNA767368 https://www.ncbi.nlm.nih.gov/bioproject/PRJNA767368/ (human RNAseq) [34], PRJNA768678 https://www.ncbi.nlm.nih.gov/bioproject/PRJNA768678/ (human 16S) [35], and PRJNA768820 https://www.ncbi.nlm.nih.gov/bioproject/PRJNA768820/ (FMT 16S) [36].

## Abbreviations

NSCLC: Non-small cell lung cancer
ICI: Immune checkpoint inhibitor/inhibition
PD-1: Programmed cell death receptor 1
PD-L1: Programmed cell death ligand 1
MCC: Moffitt Cancer Center
CTLA-4: Cytotoxic T-lymphocyte associated protein 4
PFS: Progression-free survival
ASV: Amplicon sequence variant
LDTM: Liquid Dental Transport Medium
GF-WT: Germ-free wild-type
LLC: Lewis lung carcinoma
IVIS: InVivo Imaging System
R: Responder
NR: Non-responder
TAM: Tumor-associated macrophage
PCoA: Principal coordinates analysis
PCA: Principal component analysis
EPQ: Electronic Patient Questionnaire
IACUC: Institutional Animal Care and Use Committee
UF: University of Florida
FMT: Fecal microbiota transplantation
QIIME2: Quantitative Insights into Microbial Ecology version 2
PERMANOVA: Pmutational multivariate analysis of variance
