# Supplementary figures and images for: Interaction of bacterial genera associated with therapeutic response to immune checkpoint PD-1 blockade in a United States cohort
Source: Genome Med. 2022 Mar 29;14:35. doi: 10.1186/s13073-022-01037-7 (PMC8961902; doi:10.1186/s13073-022-01037-7)

Figure S1

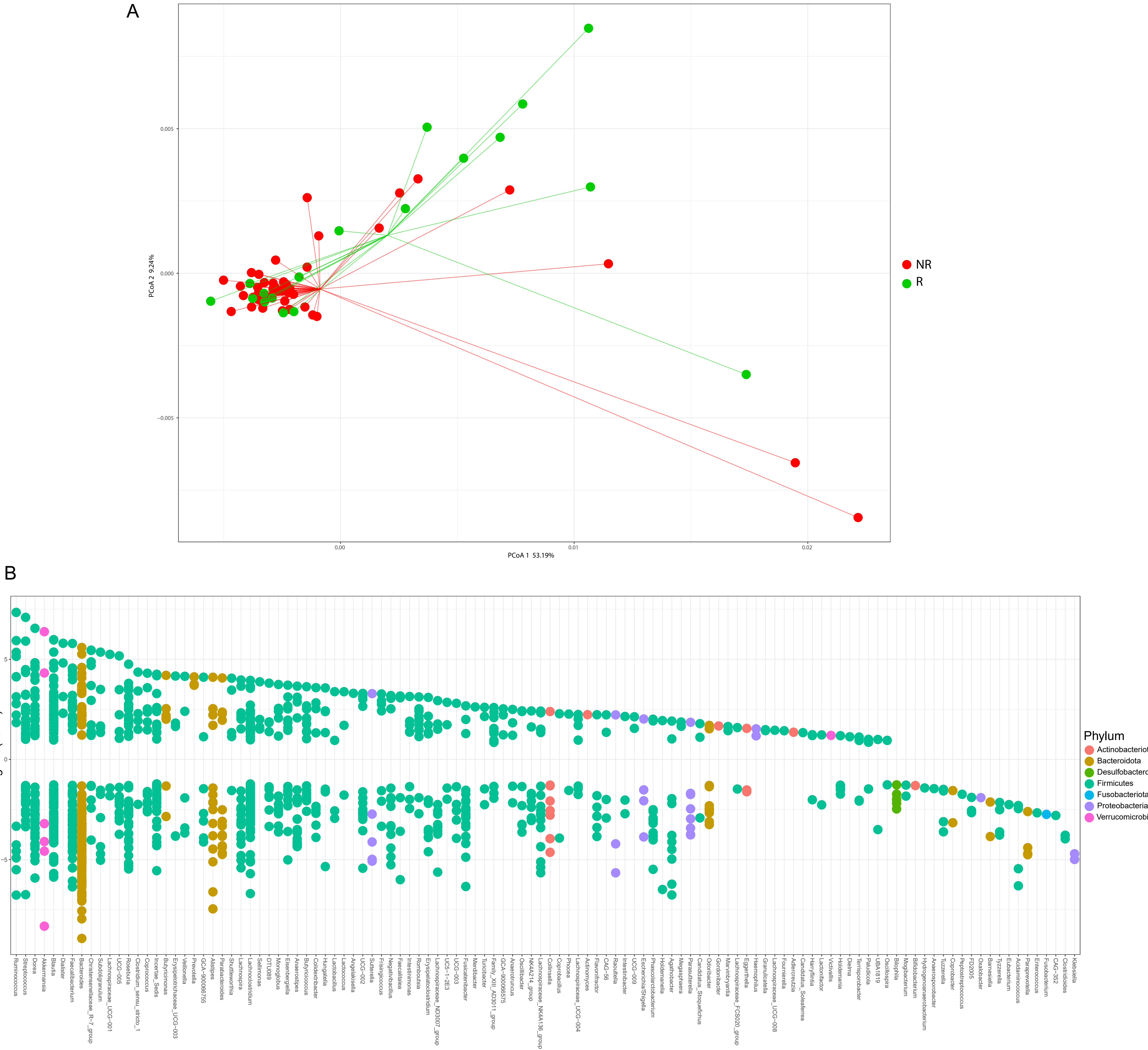

Supplement: Supplementary file 1 — Additional file 1: Fig. S1. PCoA of baseline responder versus non-responder subjects, and full log fold change plot of all significantly enriched amplicon sequence variants (ASVs). [file 13073_2022_1037_MOESM1_ESM.pdf]

Figure S2

A

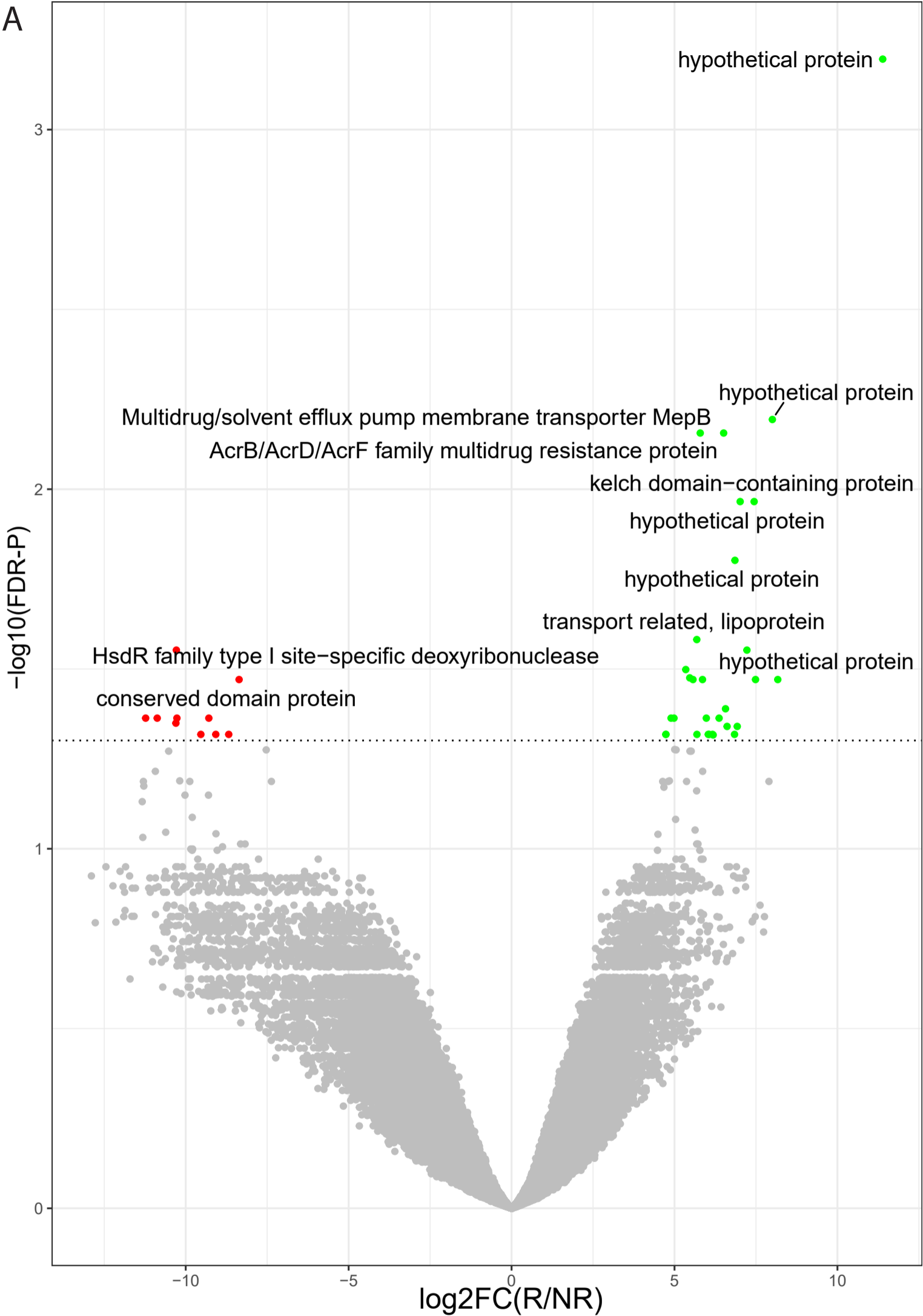

B

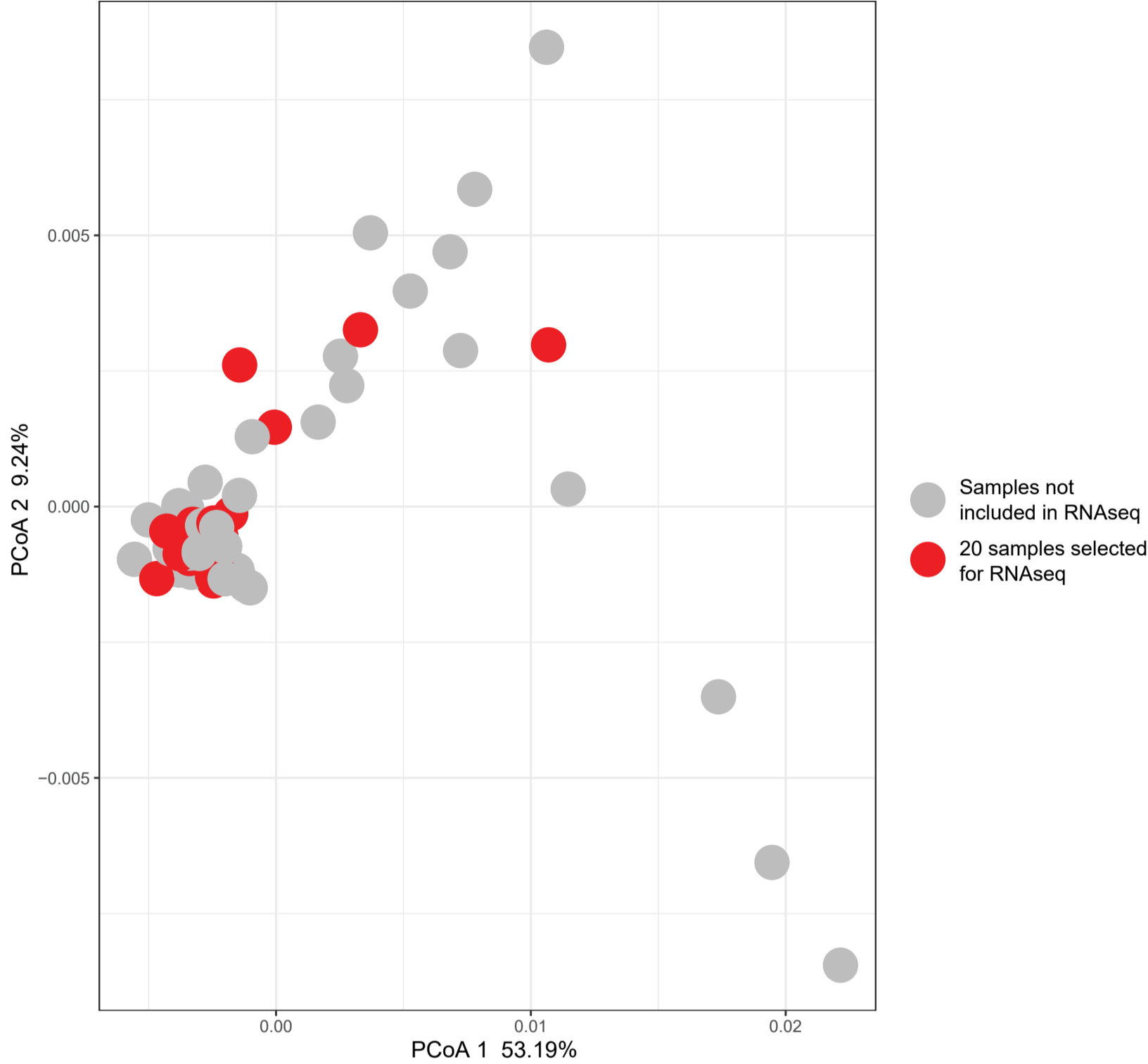

C

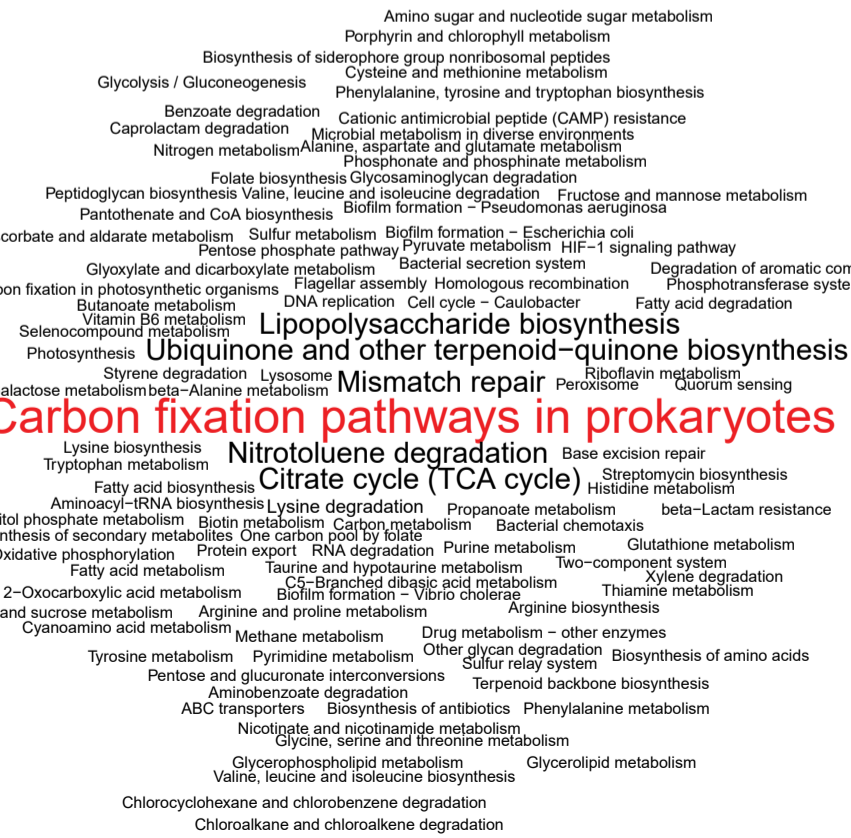

D

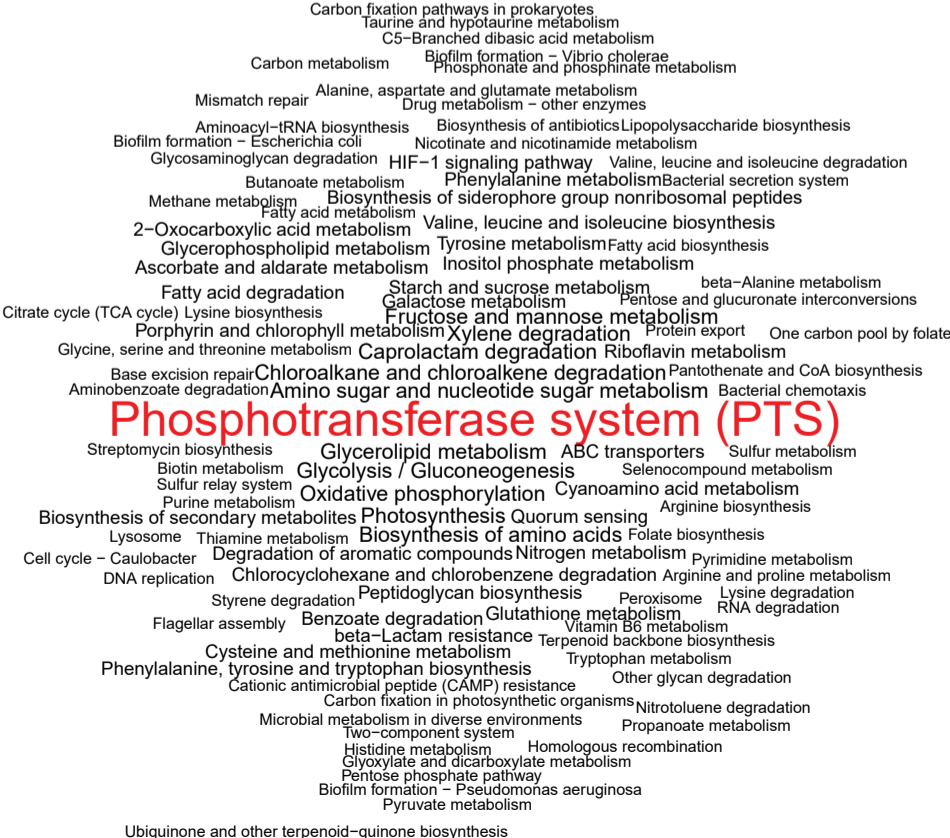

Significant ● Enriched in NR and FDR < 0.05 ● Enriched in R and FDR < 0.05 ● FDR >= 0.05

Supplement: Supplementary file 2 — Additional file 2: Fig. S2. PCoA of RNAseq samples, Volcano plot visualization of gene expression for R versus NR subjects, and word cloud representation of functional pathways enriched in R and NR subjects. [file 13073_2022_1037_MOESM2_ESM.pdf]

# Figure S3

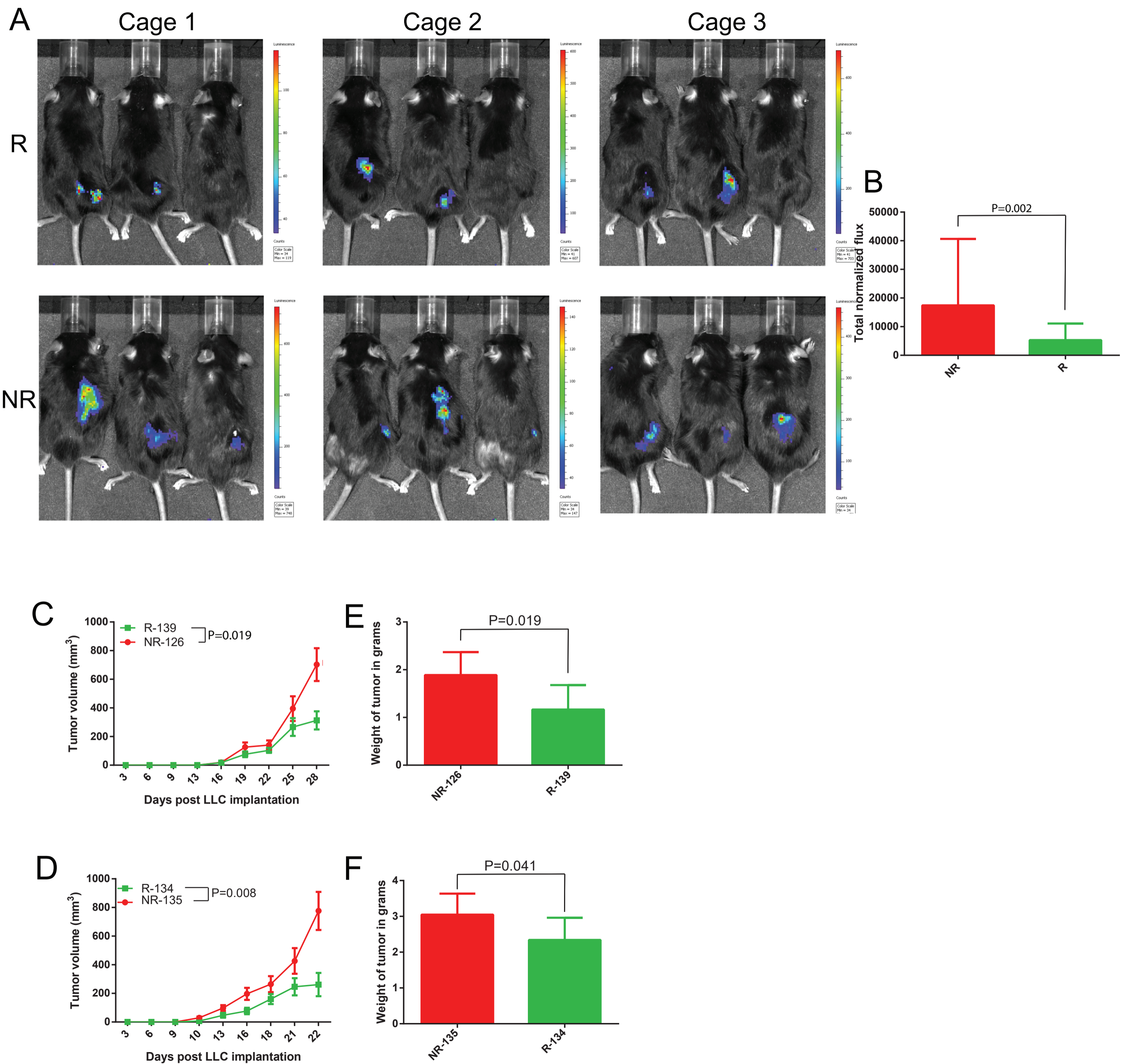

Supplement: Supplementary file 3 — Additional file 3: Fig. S3. Endpoint IVIS imaging and quantification and single donor FMT experiment tumor growth. [file 13073_2022_1037_MOESM3_ESM.pdf]

Figure S4

A

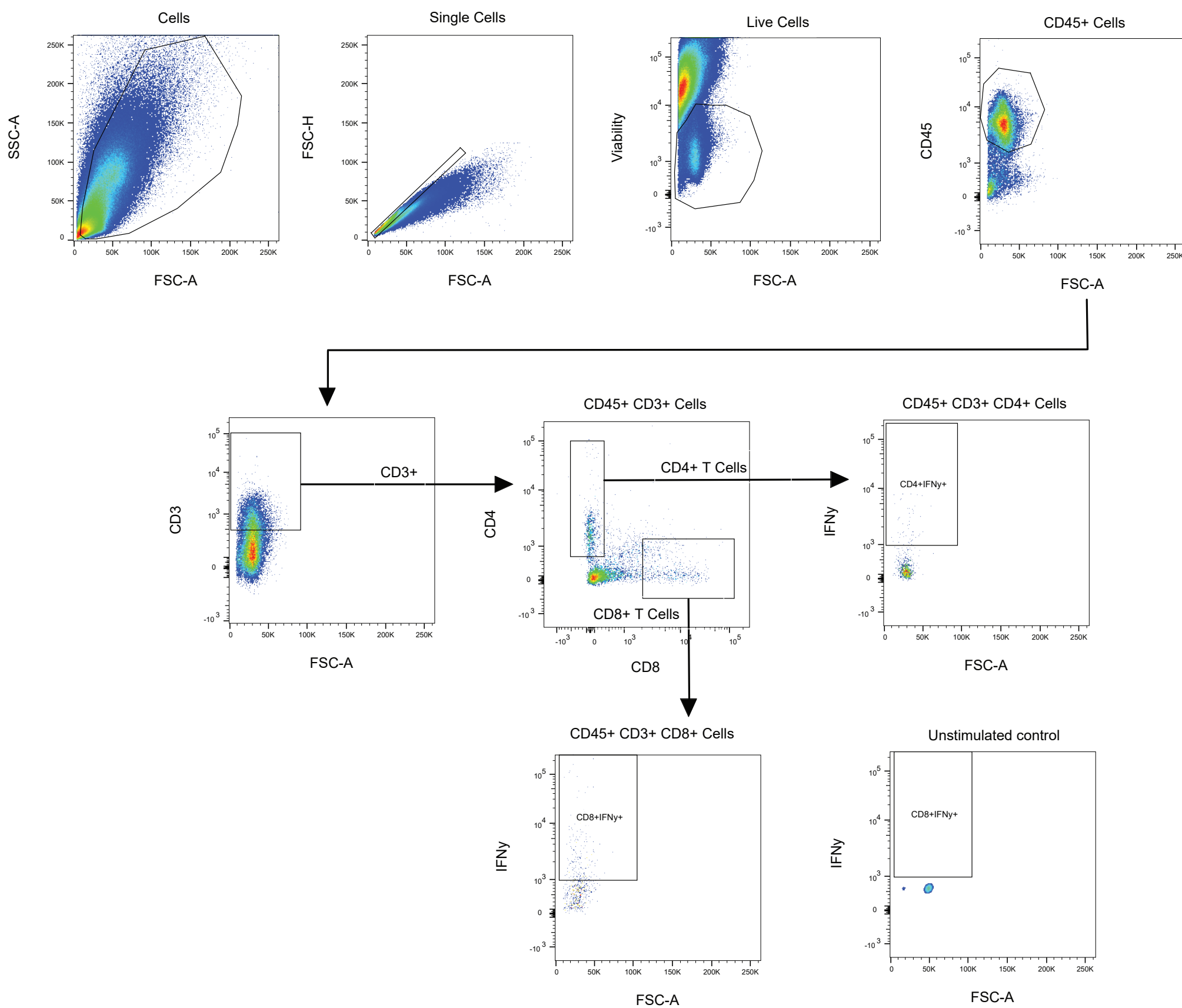

B

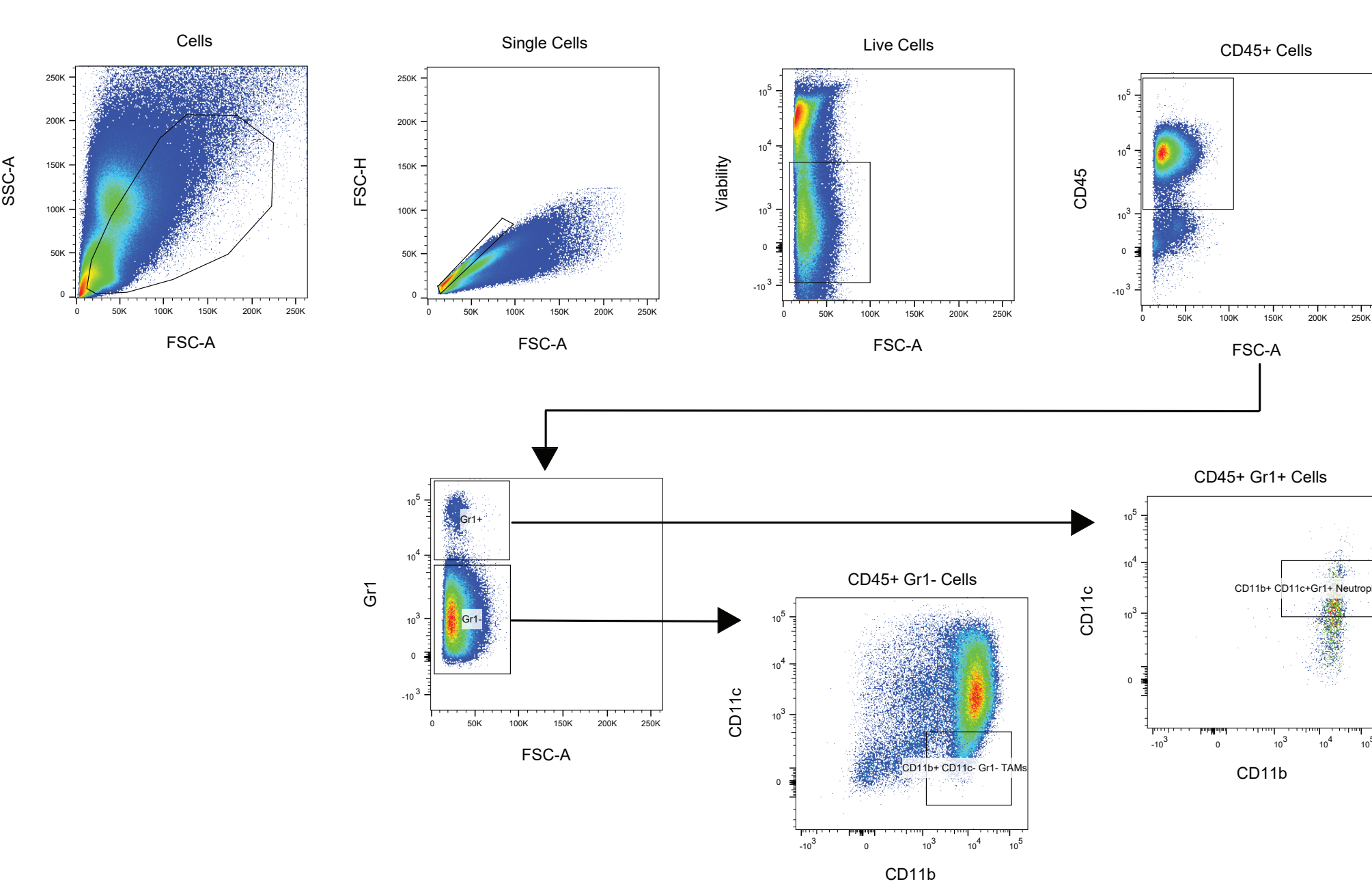

C

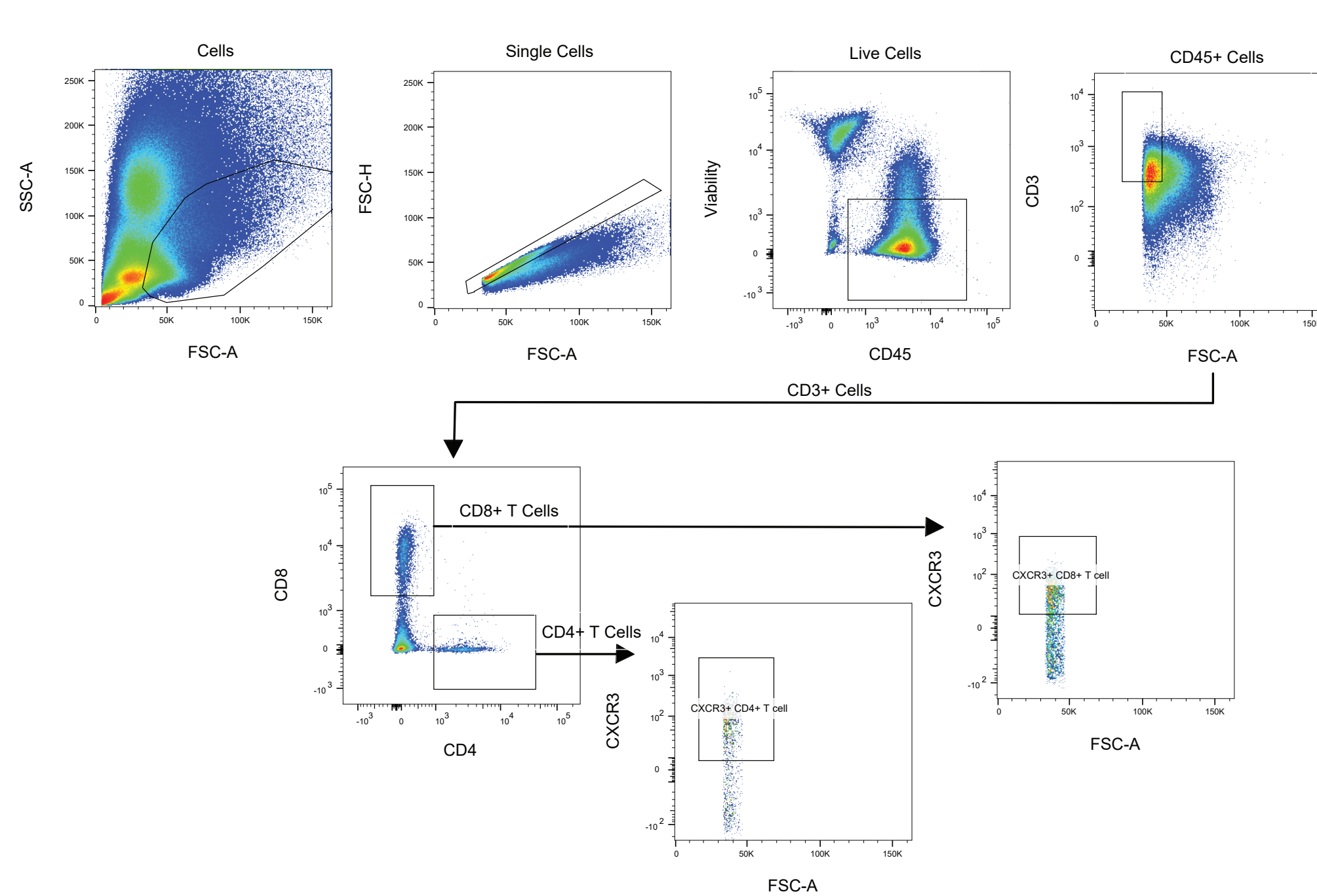

Supplement: Supplementary file 4 — Additional file 4: Fig. S4. Representative flow cytometry gating strategies. [file 13073_2022_1037_MOESM4_ESM.pdf]

# Figure S5

## A

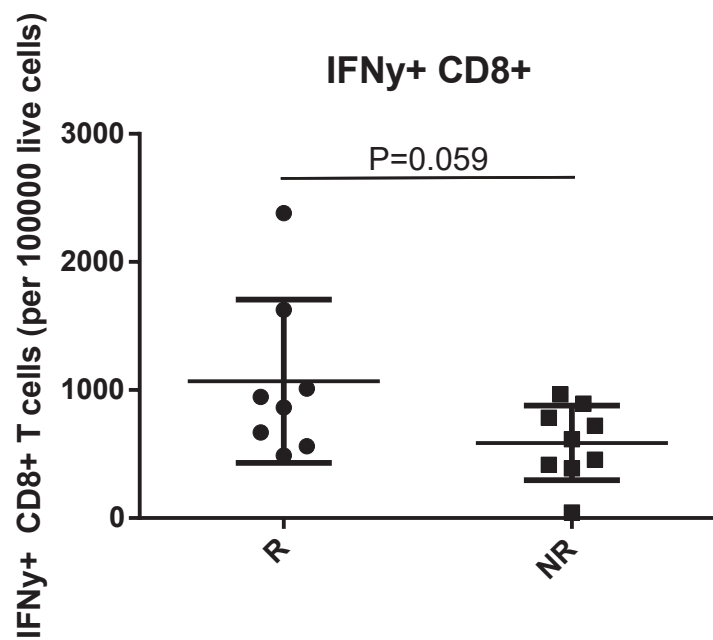

## B

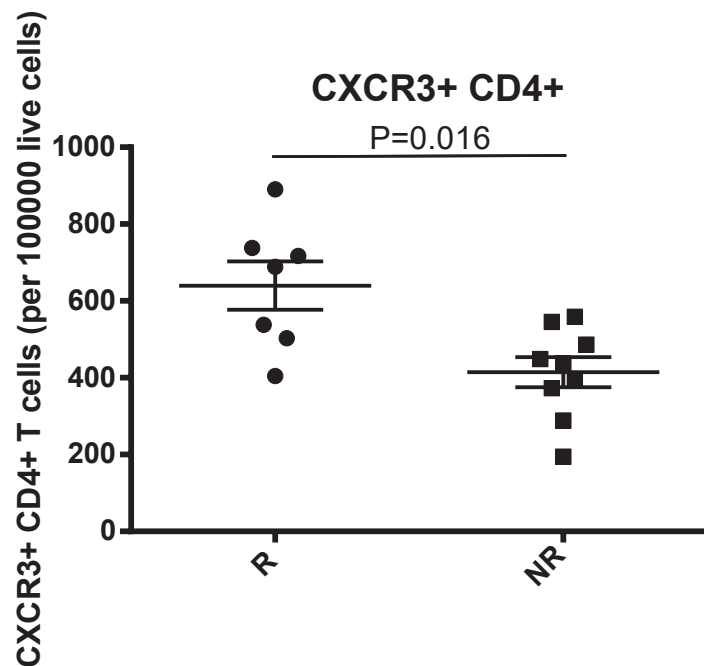

## C

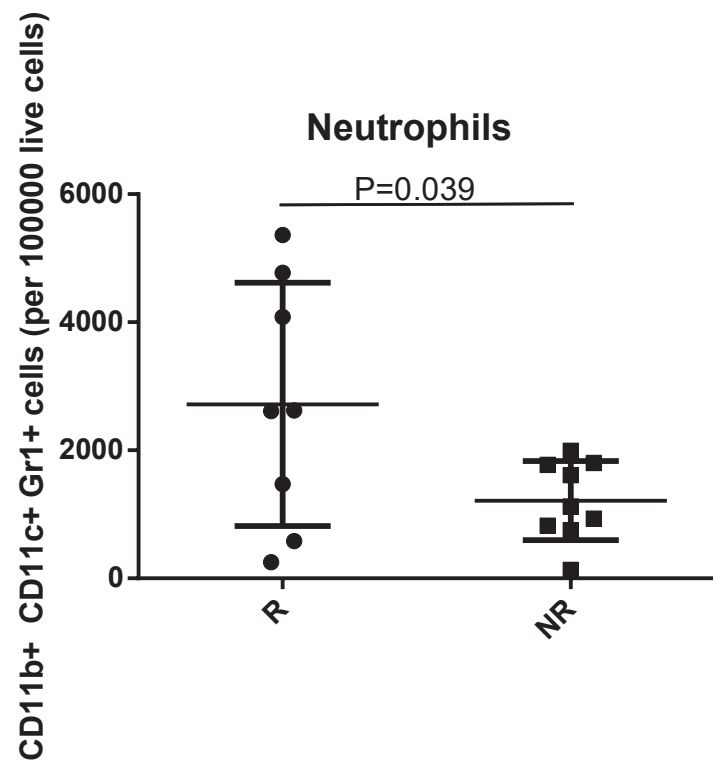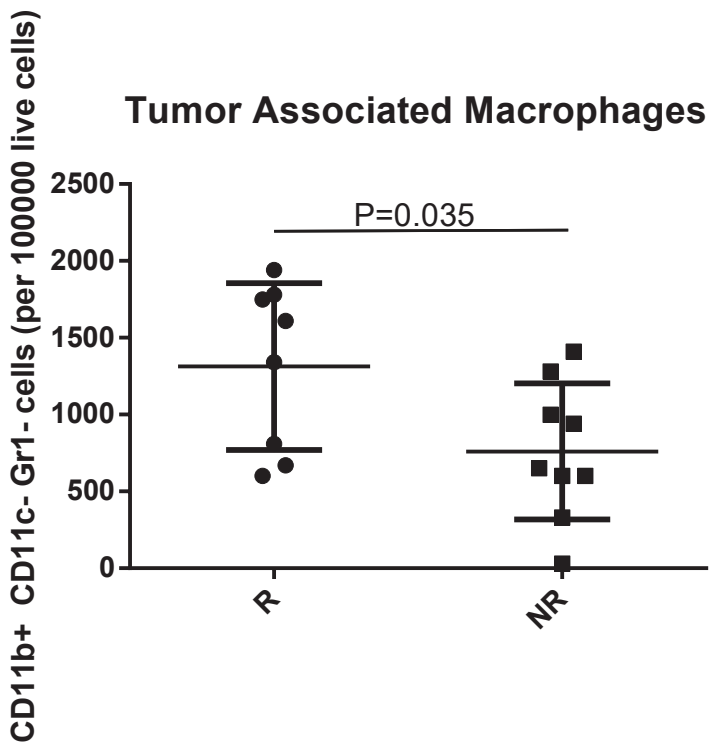

Supplement: Supplementary file 5 — Additional file 5: Fig. S5. Quantitative representation of flow cytometric analysis. [file 13073_2022_1037_MOESM5_ESM.pdf]
