# Supplementary material for: Interaction of bacterial genera associated with therapeutic response to immune checkpoint PD-1 blockade in a United States cohort
Source: Genome Med. 2022 Mar 29;14:35. doi: 10.1186/s13073-022-01037-7 (PMC8961902; doi:10.1186/s13073-022-01037-7)
Supplement: Supplementary file 7 — Additional file 7. Supplementary Figure Legends. [file 13073_2022_1037_MOESM7_ESM.docx]

**SUPPLEMENTARY LEGENDS**

**Supplementary Figure 1.** (A) Weighted UniFrac Principal coordinates analysis (PCoA) of baseline samples from human subjects showing responders (n=18) versus nonresponders (n=47). (B) Log_2_ fold change (log2FC) plot of all significantly (FDR-P < 0.05) enriched amplicon sequence variants (ASVs) in responder and non-responder subjects. Filled circles located above y=0 indicate enrichment in responders and those below indicates enrichment in non-responders.

**Supplementary Figure 2.** (A) Weighted UniFrac Principal coordinates analysis (PCoA) of baseline samples from human subjects showing samples selected for RNA-seq analysis (n=20, red filled circles) and those not selected (n=45, gray filled circles). (B) Volcano plot of metatranscriptomic data displaying the pattern of gene expression for responders (R) versus non-responders (NR). Significantly differentially expressed genes (FDR-P < 0.05) that are enriched in non-responders are shown in red, significantly differentially expressed genes (FDR-P < 0.05) that are enriched in responders are shown in green and genes with FDR-P ≥ 0.05 are shown in gray. The black dotted horizontal line represents FDR-P = 0.05. Selected genes that are enriched in responders or non-respondersare are indicated on the graph. Word cloud representation of enriched KEGG Ortholog (KO) functional profiles pathways constructed from microbial RNA sequencing showing metabolic pathways enriched in (C) responders and (D) non-responders. Pathways in red font are significantly enriched FDR-P < 0.05. Font size corresponds to -log_10_(FDR P).

**Supplementary Figure 3.** (A) Endpoint IVIS imaging via bioluminescence measurement of luciferase activity of LLC-luc implanted mice colonized with human pooled responder (n=9) and non-responder (n=9) inoculum following treatment with anti-PD-1 monoclonal antibody, separated by cage. (B) Quantification of region of interest (ROI) of bioluminescent signal per mouse by IVIS imaging software represented by mean ± SD of total normalized flux of responder (n=9) and non-responder (n=9) mice. Mann Whitney P=0.002 (C) Growth curve of LLC-luc subcutaneous allograft tumors after human fecal microbiota transplant from single donor R-139 or NR-126 into germ-free mice (n=9/group) treated with anti-PD-1 monoclonal antibody injection. Each point is tumor volume mean ± SEM. ANOVA P=0.019 at endpoint (D) Growth curve of LLC-luc subcutaneous allograft tumors after human fecal microbiota transplant from single donor R-134 or NR-135 into germ-free mice (n=9/group) treated with anti-PD-1 monoclonal antibody injection. Each point is tumor volume mean ± SEM. ANOVA P=0.008 at endpoint (E) Mean ± SEM of tumor weight at endpoint after human fecal microbiota transplant from single donor R-139 or NR-126 into germ-free mice (n=9/group) treated with anti-PD-1 monoclonal antibody injection. Mann Whitney P=0.019 (F) Mean ± SEM of tumor weight at endpoint after human fecal microbiota transplant from single donor R-134 or NR-135 into germ-free mice (n=9/group) treated with anti-PD-1 monoclonal antibody injection. Mann Whitney P=0.041.

**Supplementary Figure 4.** (A) Representative flow cytometry gating strategy used for T cell IFNγ analysis (B) Representative flow cytometry gating strategy used for tumoral myeloid population panel (C) Representative flow cytometry gating strategy used for broad T cell activation panel.

**Supplementary Figure 5.** Quantitative representation of flow cytometric analysis for: (A) number of CD8+ IFNγ+ T cells per 100,000 live single tumor cells in responder (n=8) and non-responder (n=9) tumors. Mann Whitney P=0.059.; (B) number of CD4+ CXCR3+ T cells per 100,000 live single tumor cells in responder (n=7) and non-responder (n=9) tumors. Mann Whitney P=0.016; (C) number of neutrophils (Gr1+ CD11c+ CD11b+ cells) per 100,000 live single tumor cells in responder (n=8) and non-responder (n=9) tumors. Mann Whitney P=0.039; (D) number of macrophages (Gr1- CD11c- CD11b+ cells) per 100,000 live single tumor cells in responder (n=8) and non-responder (n=9) tumors. Mann Whitney P=0.035.

**Supplementary Table 1.** Significantly upregulated pathways in responder and non-responder human subjects by RNAseq analysis.
